# Editorial: Neural bases of binocular vision and coordination and their implications in visual training programs

**DOI:** 10.3389/fnint.2015.00047

**Published:** 2015-08-13

**Authors:** Olivier A. Coubard

**Affiliations:** The Neuropsychological Laboratory, CNS-FedParis, France

**Keywords:** binocular vision, eye movements, visual pathways, neurovisual disorders, visual rehabilitation

## Opening

To see or not to see? That is the question of this research topic. How do human beings see not with their eyes but with their brain, which lies in a moving body, itself evolving in a continuously changing environment? What and how do humans see in the context of a particular task at a given moment? How do humans cease to see after some damage in the brain or neurofunctional disorder? And how may the basic science of eye movements and vision help to develop efficient visual training programs?

The present research topic, entitled *Neural bases of binocular vision and coordination and their implications in visual training programs*, aims at putting forward our knowledge of the neural underpinnings of vision in its motor, sensory, cognitive, emotional, and vegetative expressions. It does not target an exhaustive collection of what we know in the field of visual neurosciences. For that purpose, the reader may refer to the volume sets by Chalupa and Werner (2003). Rather, this research topic focuses on the latest findings on the neural aspects of eye movements and visual perception that directly help to understand and improve visual training programs in pathological conditions. Such disorders follow damages of the cerebral visual pathways (e.g., hemianopia) or refer to syndromes hitherto believed to be peripheral but in which neurophysiology and brain imaging are uncovering neural correlates or causes (e.g., amblyopia).

The research topic is divided into three parts respectively dedicated to eye movements, visual perception, and visual training programs, each having six chapters, and starts with an overview. In the introductory chapter, Coubard, Urbanski, Bourlon and Gaumet (Coubard et al., 2014) remind the reader of the importance of action in visual processing before describing the cascade of physiological mechanisms underlying eye movements, followed by a description of the five main neurovisual systems. After an overview of pathological conditions causing not eye but brain blindness—also called neurovisual disorders—the authors end by describing the disciplines of visual rehabilitation.

## Part I—neural bases of eye movements and binocular coordination

The important role of fixational eye movements in binocular vision is developed by Otero-Millan, Macknik and Martinez-Conde (Otero-Millan et al., 2014). The authors review the past 50 years of research on binocular coordination of each fixation eye movement type and how pathologies of binocular vision impact such coordination. Percheron, François and Pouget (Percheron et al., 2015) challenge histological, anatomical, and functional definitions of the frontal eye field (FEF) in primate species—a critical area in eye movement control subjected to passionate debates. This review will certainly help redefining new methodologies and thinking paradigms for future research. The role of prefrontal neurons, particularly of the dorsolateral prefrontal cortex (DLPFC), is reviewed by Funahashi (2014). The author describes prefrontal neuron pre-saccadic and post-saccadic memory-related activities, and offers insightful perspectives about attentional control processes such as working memory updating and the control of performance. The impact of visual pathology like amblyopia on saccadic behavior is addressed in an original study by Perdziak, Witkowska, Gryncewicz, Przekoracka-Krawczyk and Ober (Perdziak et al., 2014). Using a delayed saccade task, the authors show that the amblyopic eye is slower to respond than the non-amblyopic one. In dyslexia, eye movement behavior and visual search ability are explored by Seassau, Gérard, Bui Quoc and Bucci (Seassau et al., 2014). The authors show that oculomotor control in reading and binocular coordination of saccades in visual search are impaired in dyslexic children as compared to typical readers. Finally, Noorani (2014) reviews 34 years of research on the LATER (linear approach to threshold with ergodic rate) physiological model. The author points out that saccadic behavior, even in advanced decision tasks such as antisaccade and sequential decisions, can be accurately predicted by the model.

## Part II—neural bases of visual perception and binocular vision

To examine visual pathologies, Raz and Levin (2014) recommend using functional magnetic resonance imaging (fMRI) and Diffusion Tensor Imaging (DTI). Illustrating damages at different levels of the visual pathways, the authors show the advantages of combining the topological and hodological approaches to understand visual phenomena. Strabismus has until recently been considered only as a peripheral visual pathology. Bui Quoc and Milleret (2014) show how vision science and clinical ophthalmology are now advanced enough to comprehend possible cerebral origins of strabismus, whose complexity mirrors that of its polymorphism and various expressions. Amblyopia, deriving from abnormal visual experience (strabismus, anisometropia), has also been preferentially examined through the ophthalmological lens. Joly and Frankó (2014) review neuroscientific discoveries in amblyopia and the way functional brain imaging helps to map deficits in 3D vision networks within occipital-parietal and occipital-temporal pathways. Visual scene perception has been subjected to major advances for the last three decades. Kauffmann, Ramanoël and Peyrin (Kauffmann et al., 2014) describe that scenes are processed in terms of low/high spatial frequencies activating occipital areas in relation to peripheral/foveal representations and specific areas within the occipital-temporal cortex. In an original study using a saccade choice task, Boucart, Calais, Lenoble, Moroni and Pasquier (Boucart et al., 2014) show that participants suffering from posterior cortical atrophy compared to participants with Alzheimer's disease are impaired in detecting targets in a scene and do not benefit from contextual information. Vision is also a major issue in psychiatry. Notredame, Pins, Deneve and Jardri (Notredame et al., 2014) review psychophysical and neurophysiological findings about visual perception in schizophrenia. Through illusory paradigms and Bayesian inference framework, they explain perceptual changes in schizophrenia providing insightful directions to study its neural mechanisms.

## Part III—visual training programs in organic deficits and their neural bases

Taub, Mark and Uswatte (Taub et al., 2014) examine the possibility that techniques of Constraint Induced (CI) therapy previously employed in the rehabilitation of movement may prove useful for the treatment of visual deficits. The strengthening of diminished neural connections is developed to account for CI-induced neurovisual improvements. Homonymous hemianopia (HH) is a symmetric loss of vision in both eyes following unilateral retrochiasmatic lesion. Perez and Chokron (2014) review the different visual rehabilitation techniques in HH and the potentials of blindsight, the implicit visual function enabling hemianopic patients of performance escaping their consciousness. Describing the neural mechanisms of plasticity and reorganization after brain damage and those induced by visual training programs is a challenging issue, addressed by Urbanski, Coubard and Bourlon (Urbanski et al., 2014), suggesting that future research cannot dispense with studying the cerebral changes in relationship to behavioral improvements. In an original study, Alvarez, Jaswal, Gohel and Biswal (Alvarez et al., 2014) measured brain changes induced by vergence training in participants with convergence insufficiency. They demonstrate that the vergence peak velocity correlates with blood-oxygen-level dependent (BOLD) signal changes within the FEF, posterior parietal cortex and cerebellar vermis. Non-invasive manipulation techniques have become interesting tools to modulate vision in humans. Vernet, Quentin, Chanes, Mitsumasu and Valero-Cabré (Vernet et al., 2014) review the malleability of regions involved in eye movements and visual perception, particularly the FEF, and extend to the therapeutic interest of these modulatory techniques. In psychiatry, a study by Beynel, Chauvin, Guyader, Harquel, Szekely, Bougerol and Marendaz (Beynel et al., 2014) explores the effects of DLPFC intermittent theta burst stimulation in participants suffering from bipolar disorder. They show that neuromodulation improves performance in the antisaccade task, which also correlates with mood changes.

## Closure

In a world where the human factor introduces unlimited degrees of complexity, science has an ability to focus the attention of scientists and clinicians on restricted objects and questions. It does it over time, worldwide, disregarding daily life contingencies, keeping ideas strong and emotions hot. To see or not to see was the question of the research topic (see Section Opening). To address it, 59 authors from six countries—France, Israel, Japan, Poland, UK and USA—have generously accepted to contribute so as to put forth our knowledge of the neural underpinnings of eye movements and visual perception. Thirty-six other scientists from sixteen countries—Brazil, Canada, China, Finland, France, Germany, Hungary, Israel, Italy, Netherlands, Poland, Portugal, Spain, Switzerland, UK, and USA—have also offered their expertise to challenge authors within the limits that scientific exercise imposes. The outcome of this synergy is the object the reader now holds before his/her eyes. There is no doubt that this research topic will inspire both scientists and clinicians investigating and helping patients in the field of neurovision for the next 10 years.

## Conflict of interest statement

The author declares that the research was conducted in the absence of any commercial or financial relationships that could be construed as a potential conflict of interest.
